# Spontaneous Isolated Visceral Artery Dissection in a Middle Aged Male

**DOI:** 10.1155/2017/3704348

**Published:** 2017-01-01

**Authors:** Eric Melnychuk, Robert Strony

**Affiliations:** Department of Emergency Medicine, Geisinger Medical Center, 100 North Academy Avenue, Danville, PA 17822, USA

## Abstract

Epigastric pain is a common complaint made by patients being evaluated in the emergency department. Spontaneous isolated visceral artery dissection is a rare cause with no reported prevalence. We present a case of a 37-year-old male evaluated in the emergency department for epigastric pain and subsequently diagnosed with a spontaneous isolated celiac artery dissection with involvement of the hepatic and splenic arteries. Recent case series suggest this disease may be managed medically in most cases. Surgical intervention may be considered for significant bleeding or signs of intestinal ischemia.

## 1. Case Report

**Figure 1:**
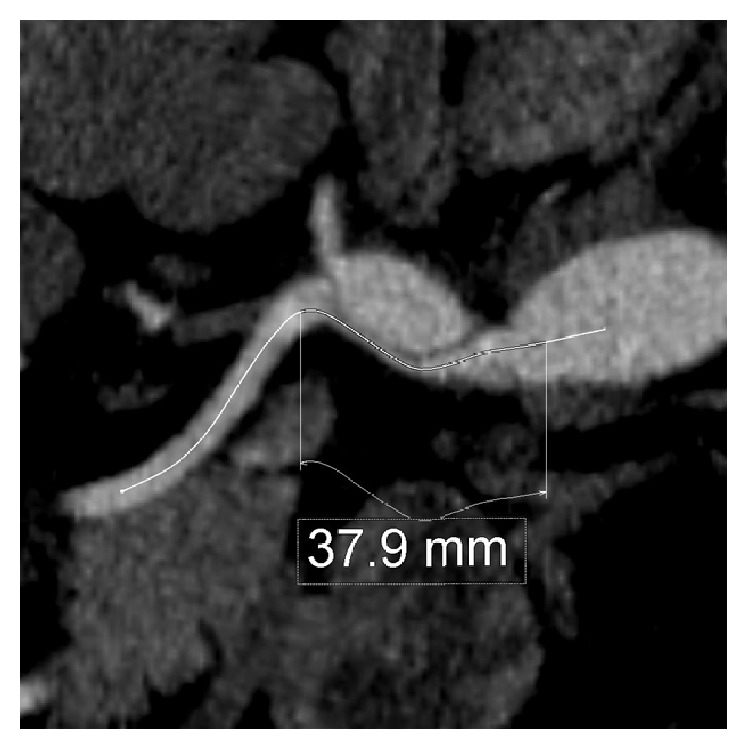
CT angiogram showing length of dissection of the celiac artery to the splenic vein.

**Figure 2:**
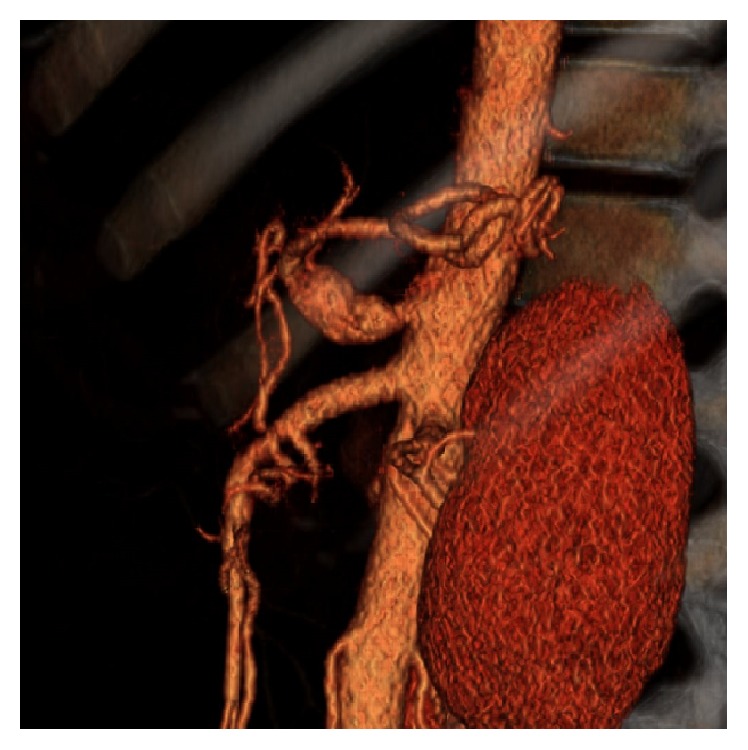
CT angiogram reconstruction of the celiac artery at the time of diagnosis.

A 37-year-old Caucasian male presented to an emergency department with worsening epigastric pain of 4-day duration. He stated that his pain initially began as indigestion while he was leaning forward getting out of bed and progressed to a constant aching pain with associated nausea. He stated that bending forward exacerbated the pain. He denied prandial pain, melena, hematochezia, or recent change in bowel habits. History was notable for hypertension which was being managed without need for antihypertensive medications. Surgical history was not significant for any prior abdominal surgeries. His social history was significant for tobacco abuse which consisted of a half pack of cigarettes a day for 25 years. Initial vital signs were significant for hypertension. Physical exam on presentation was significant for minimal epigastric tenderness with normoactive bowel sounds without rebound or guarding. Lab analysis was not significant for elevated lipase, liver function abnormalities, anemia, or electrolyte abnormalities. Computed tomography (CT) of the abdomen and pelvis with IV contrast was performed which showed a filling defect in the celiac artery. Due to concern for dissection, a CT angiogram of the abdomen and pelvis was performed. The CT angiogram showed a 40.1 mm dissection from the celiac origin into the hepatic artery as well as a 37.9 mm dissection from the celiac origin into the splenic artery (Figures 1 and 2). He was also found to have a beaded-appearing left main renal artery, raising concerns for fibromuscular dysplasia.

The patient was admitted for observation, given 1000 mL unfractionated heparin bolus, and started on an unfractionated heparin infusion. The patient was evaluated by vascular surgeons and gastroenterologists. When given the choice for stenting the lesion for symptomatic control, he elected not to have the lesion stented. Prior to discharge he was started on 81 mg aspirin daily and a follow-up appointment with repeat CT angiography was scheduled in 2 months. Follow-up CT angiogram two months after his initial diagnosis showed minimal enlargement of the dissection. At that time during his vascular surgery follow-up, the patient stated improvement in his abdominal pain. At that time he was scheduled for a 6-month appointment and follow-up CTA.

## 2. Discussion

Visceral artery dissection typically occurs as a complication of an extension of a descending aortic dissection. However, spontaneous isolated visceral artery dissection has been described in the literature. The prevalence and etiology of this disease are unknown as it is uncommon and the literature consists only of case reports and case series. Spontaneous dissection of the superior mesenteric artery is most common, followed by celiac artery dissection [1–3]. Patient characteristics typically include male gender, age over 50, hypertension, and history of smoking. However, spontaneous celiac artery dissection has been reported in younger, healthy patients [4]. Treatment is not well defined and is currently tailored to the patient's severity of dissection and risk of progression [4, 5]. Observation with or without anticoagulation and close follow-up, including serial imaging studies, may be effective for patients without signs of intestinal infarction or bleeding [3].

The most common sign of spontaneous visceral artery dissection is acute onset epigastric or abdominal pain not preceded by trauma. Associated symptoms may include nausea, vomiting, or diarrhea [6–8]. Patients may have tenderness to abdominal palpation but usually do not exhibit peritoneal signs. Contrast-enhanced abdominal CT is the imaging modality of choice and the most common imaging findings are presence of an intimal flap, false lumen thrombosis, or aneurysmal dilatation [2, 9]. Misdiagnosis can occur even with contrast-enhanced CT [10]. Radiological classification for further characterization of SMA dissections was reported in 2007 and included presence of a false lumen with or without reentry into the true lumen and thrombosis of the false lumen with or without a blood-filled pouch bulging from the true lumen into a thrombosed false lumen [8]. This classification system has subsequently been used for characterization of celiac artery dissections as well.

Complications of visceral arterial dissection include intestinal ischemia or infarction from stenosis of the true lumen, aneurysm formation due to vessel wall degeneration, and vessel rupture. Initial conservative medial management with anticoagulation and close follow-up can be applied in the absence of bowel ischemia or vessel rupture. Anticoagulation is thought to prevent vessel thrombosis and embolization. Surgical management with endovascular stenting can be used in patients with persistent abdominal pain. Open surgical management is rare and is typically used when the dissected vessel has ruptured.
